# A new web-based framework development for fuzzy multi-criteria group decision-making

**DOI:** 10.1186/s40064-016-2198-1

**Published:** 2016-05-11

**Authors:** Mohamed Hanine, Omar Boutkhoum, Abdessadek Tikniouine, Tarik Agouti

**Affiliations:** Laboratory of Engineering and Information Systems, Department of Computer Science, Faculty of Sciences Semlalia, Cadi Ayyad University, Marrakech, Morocco; Team of Telecommunications and Computer Networks, Faculty of Sciences Semlalia, Cadi Ayyad University, Marrakech, Morocco

**Keywords:** Fuzzy multi-criteria group decision-making (FMCGDM), Fuzzy logic, Web-based framework, Fuzzy Delphi, Fuzzy AHP, Fuzzy TOPSIS

## Abstract

Fuzzy multi-criteria group decision making (FMCGDM) process is usually used when a group of decision-makers faces imprecise data or linguistic variables to solve the problems. However, this process contains many methods that require many time-consuming calculations depending on the number of criteria, alternatives and decision-makers in order to reach the optimal solution. In this study, a web-based FMCGDM framework that offers decision-makers a fast and reliable response service is proposed. The proposed framework includes commonly used tools for multi-criteria decision-making problems such as fuzzy Delphi, fuzzy AHP and fuzzy TOPSIS methods. The integration of these methods enables taking advantages of the strengths and complements each method’s weakness. Finally, a case study of location selection for landfill waste in Morocco is performed to demonstrate how this framework can facilitate decision-making process. The results demonstrate that the proposed framework can successfully accomplish the goal of this study.

## Introduction

Multi-criteria group decision-making (MCGDM) was derived from the traditional MCDM and techniques of group decision-making. MCGDM problems arise in situations where a group of decision-makers faces a problem of selecting the optimal solution among different possible alternatives. The MCGDM is a process that contains five main steps starting with determining a group of decision-makers and ending with the selection of an alternative (Jacquet-Lagrèze and Siskos 2001).

Generally, decision-makers have to consider both quantitative and qualitative assessments of the criteria in evaluating the considered alternatives. Classical MCGDM problems usually present judgments as crisp numerical values and need to evaluate the performance of solutions according to each criterion. On the one hand, information about the alternatives is often imprecise and the decision-makers can only provide approximate, incomplete or not well-defined information. On the other hand, it is to be taken into account that some criteria may be subjective. To overcome these limitations of the classical MCGDM, many researchers concentrated on incorporating fuzzy set theory in MCGDM.

Fuzzy set theory is alternative models in cases of uncertain data or lack of knowledge. Fuzzy MCGDM (FMCGDM) is generally based on fuzziness of MCGDM theories where it is a tool that supports decision-makers to manage the uncertainty of their judgments (Arslan and Aydin 2009). Actually, when decision-makers evaluate an exact judgment by crisp numbers rather than qualitative expressions, MCGDM uses fuzzy evaluations and presents the suitability of alternatives according to each other.

Additionally, FMCGDM methodologies may help resolve various problems frequently encountered in group decision-making. It principally aims to reduce the effects of ambiguity like human judgment and preferences while searching for the appropriate solution (Słowiński 1998). Wang (2009) presented an approach that integrates grey relation analysis and FMCGDM for evaluating and selecting financial performance of Taiwan container lines. Fanghua and Guanchun (2009) introduced a FMCGDM model based on weighted Borda scoring method for watershed ecological risk management. In this model, decision-makers provide an independent judgement, then, all judgements are incorporated by using their subjective/objective weights. The use of Borda scoring method is to rank the potential alternatives. One of the recent studies on FMCGDM is Chen et al. (2016), where a new FMCGDM method based on intuitionistic fuzzy sets and the evidential reasoning methodology is proposed. Similarly, Igoulalene and Benyoucef (2014) proposed a new fuzzy consensus-based possibility measure and TOPSIS approach dedicated to the plant selection problem. Recently, Igoulalene et al. (2015) proposed a novel fuzzy hybrid multi-criteria group decision making approaches for the strategic supplier selection problem. Although FMCGDM methods are applied to many decision-making problems, it should be noted that they are based on the use of fuzzy numbers for comparing and ranking the alternatives, which can lead to some problems, as the essential computations are quite time-consuming and may lead to errors. Hence, it is important to have an appropriate framework for applications of such methods. Over the past 10 years or so, a considerable research effort has been made to computerize many MCDA methods to resolve many problems (Ma et al. 2010; Cakir and Canbolat 2008; Hanine et al. 2016; Alptekin and Büyüközkan 2011). Cakir and Canbolat (2008) suggest that most of MCDA applications focused on the technical aspects of integration to address the analytical structure of problems. Many of those systems do not assist practitioners/researchers in choosing the optimal alternatives but instead provide decision support by facilitating information access and visualization. In addition, most available systems have been custom-built for either specific applications or data, or mono-users. Therefore, no generic free prototype can accept the judgements and preferences of group decision-makers under consideration of fuzzy systems online. For this reason, a web-based framework is developed to enable the application of three special well-known FMCGDM methods such as fuzzy Delphi, fuzzy AHP and fuzzy TOPSIS. The integration of these methods facilitates the decision-making process by allowing decision-makers to explore different aspects of a decision problem and express their judgments and preferences. FMCGDM provides a mechanism for expressing the decision-makers’ preferences and objectives for generating an optimal solution. Furthermore, FMCGDM can offer a structured environment for investigating the intensity and sources of conflicts among different decision-makers.

The application of the problem that is considered for applying and testing the framework is of environmental origin. The importance of such problems and their nature will be briefly pointed out. In environmental domain, decision-making is frequently related to waste management among others. Especially, the problem that is considered in this study is the landfill waste location selection problem. The fact that selection of the site for landfill of wastes is a fundamental activity for each region. Having an impact on the environment requires analyses to choose the most optimal location for landfill waste. Hence, the region that is considered in this case study is Casablanca, which is the most populated and important industrial region in Morocco where there exists four candidate locations for landfill wastes.

In this paper, we propose the use of fuzzy Delphi, fuzzy AHP and fuzzy TOPSIS methodology for the multi-criteria decision-making problem. In this respect, the aim of using the fuzzy Delphi is to manage a group of decision-makers for evaluating and assessing the criteria and alternatives via questionnaires. The fuzzy-AHP is used as a method to structure the problem and obtain the weights of selected criteria by incorporating the uncertainty values. Then, the fuzzy TOPSIS is taken into account to rank the available alternatives. To the best of our knowledge, this is the first paper developing a web-based framework by applying this methodology for multi-criteria decision-making problems that enables us to implement all the tasks related to the analysis in one tidy structure. The framework is implemented using PHP/Java Script Technology, MySql database, the Wamp web server and other open source software.

The rest of the paper is organized as follows: in the “MCDM systems/software” section, we discuss a survey about MCDA software/systems. In the “Methods” section, the proposed integrated methodology is concisely explained. In the “FMCGDM framework” section, we present the development of our FMCGDM framework, which will be validated by an empirical study, is illustrated in the “Application of FMCGDM framework” section. Finally, we come to end with our conclusions.

## MCDM systems/software

MCDM methods and software offer a wide spectrum of approaches to various multi-criteria problems incorporating objective values and subjective judgments. In this study, computer framework/software that implements MCDM approach is considered. Actually, free and commercial software that employ various MCDM methods and tools have been developed (Keeney and Raiffa 1976; Belton and Stewart 2002; Ishizaka and Nemery 2013). Table 1 presents some of the more popular programs divided into two categories: integrated or no-integrated approaches, also including whether the system is free or not and whether it supports the fuzzy/group models or not. Among these, the following framework/software is highlighted through their widespread use: MakeItRational, Visual PROMETHEE, and Decerns MCDA.

**Table 1:** Computer systems for decision analysis for implementation of MCDA methods (Ishizaka and Nemery 2013)

System/software	Fuzzy model	Free	Group model	MCDA methods
No-integrated approaches				
MakeItRational	No	Yes	Yes	AHP
Super decisions	No	Yes	No	ANP
RightChoice	No	Yes	No	MAUT
M-MACBETH	No	No	No	MACBETH
Visual PROMETHEE	No	Yes (limited to ten alternatives)	No	PROMETHEE I–II–III–IV–V
Electre III–IV software	No	Yes	No	Electre III–IV
Microsoft Excel solver	No	No	No	Goal programming
DEA software	No	Free and open source	No	DEA
Expert choice	No	Yes	Yes	AHP
Integrated approaches				
DecernsMCDA	Yes	No	No	AHP–MAVT–MAUT–PROMETHEE–FlowSort–TOPSIS
VISA	No	No	Yes	MAUT/MAVT

MakeItRational represents web software that contains a range of tools for the construction of the analytic hierarchy process (AHP). This software is interesting because it incorporates intuitive graphical user interfaces and provides different techniques of processing a sensitivity analysis (Ossadnik and Kaspar 2013).

As regards Visual PROMETHEE, it implements the PROMETHEE method. A number of user-friendly interfaces and graphs have certainly contributed to the success of the system and conducted sensitivity analysis to weight variation of weighting procedures. Furthermore, the GAIA (geometrical support) plan, which is the representation of a decision problem, contains all the aspects of the decision problem: the alternatives, the criteria and the decision maker’s preference information. This plan is integrated in the software to present the vector values of criteria according to alternatives (Mareschal and De Smet 2009).

Concerning Decerns MCDA (decision evaluation for complex environmental risk network systems), it aims the development of a combined, user-intuitive software platform which can use multiple data sources containing spatial and temporal data, value and judgment criteria, to offer a comprehensive risk management (Grebenkov et al. 2008). A primary prototype of the *Decerns* system, *DECERNS SDSS*, was applied for multi-criteria decision making on land-use planning and risk management with the use of Geographic Information System (GIS)-functions and one MCDA method (Yatsalo et al. 2015; Grebenkov et al. 2008).

## Methods

**Fig. 1:**
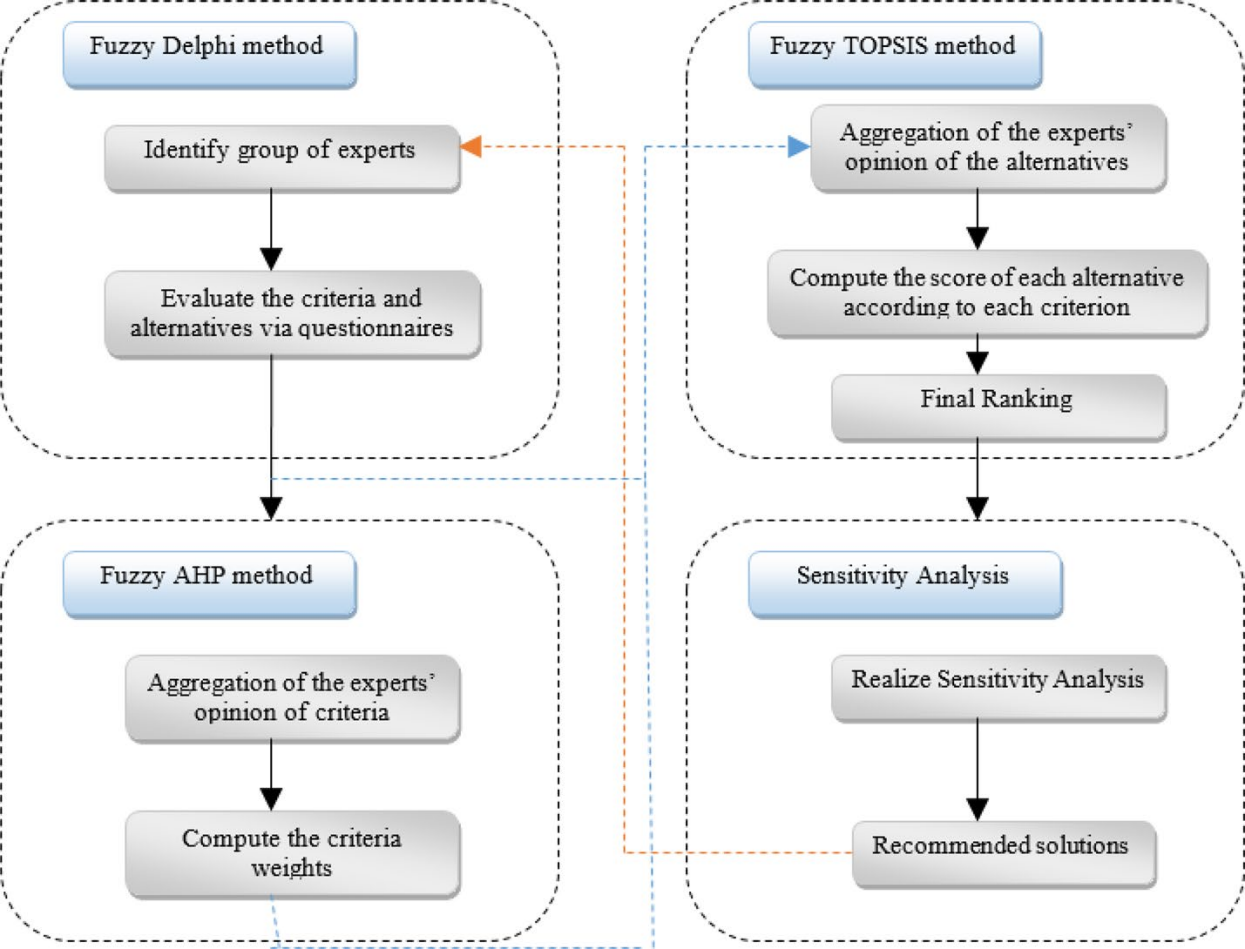
The general framework proposed for the fuzzy FMCGDM model

The proposed methodology for FMCGDM framework presented in Fig. 1, contains three methods: fuzzy Delphi, fuzzy AHP, and fuzzy TOPSIS. The model of Fig. 1 can be considered as an implementation of the FMCGDM process adjusted to the use of a decision support system/framework (e.g., FMCGDM framework) where the user can benefit from several methods to generate the appropriate decision. In this respect, the first phase is dedicated to determine the criteria with respect to the requirements (needs and wants) of the experts and decision-makers via fuzzy Delphi method (FDM). The second phase is where the weights of all the criteria and sub-criteria selected are obtained via fuzzy AHP, the output of which is then used as input to the fuzzy TOPSIS method for determining the best alternative as the third phase. Finally, the results are analyzed via sensitivity analysis. The detailed descriptions of the three phases are elaborated in the following subsections.

### Fuzzy set theory

**Fig. 2:**
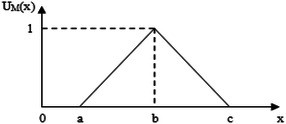
Triangular fuzzy number (TFN)

Fuzzy set theory is among the most preferred theories in decision making, which is an extension of ordinary set theory that was introduced by Zadeh (1965) for dealing with uncertainty and imprecision information associated with another. In the literature, trapezoidal and triangular fuzzy numbers (TFNs) that are the forms of fuzzy numbers are used in order to capture the vagueness of the parameters related to the topic. In this research work, TFNs are used. A triangular fuzzy number ẽ (a, b, c) will be used to consider the fuzziness of the landfill waste location selection criteria. The membership function μ(x) of the triangular fuzzy number may therefore be described as Fig. 2, and mathematical relationships (Önüt and Soner 2008; Beskese et al. 2015):

1 $$\mu_{{\tilde{e}}} (x) = \left\{ {\begin{array}{*{20}l} {0,} \hfill &\quad {x \le a} \hfill \\ {\frac{x - a}{b - a},} \hfill &\quad {a < x \le b} \hfill \\ {\frac{c - x}{c - b},} \hfill &\quad {b < x \le c} \hfill \\ {0,} \hfill &\quad {x > c} \hfill \\ \end{array} } \right.$$

The forward of fuzzy set theory used in this study are as follows:

#### **Definition 1**

Let ẽ_1_ (a_1_, b_1_, c_1_) and ẽ_2_ (a_2_, b_2_, c_2_) be two TFNs, then the vertex method is defined to calculate the distance between them as:

2 $${\text{d}}\left( {{\tilde{\text{e}}}_{1} ,{\tilde{\text{e}}}_{2} } \right) = \sqrt {\frac{1}{3}({\text{a}}_{1} - {\text{a}}_{2} )^{2} + ({\text{b}}_{1} - {\text{b}}_{2} )^{2} + ({\text{c}}_{1} - {\text{c}}_{2} )^{2} }$$

#### **Definition 2**

Let ẽ_1_ and ẽ_2_ be two TFNs. The main operations of TFNs are as follows:

3 $${\tilde{\text{e}}}_{1} \oplus {\tilde{\text{e}}}_{2} = ({\text{a}}_{1} + {\text{a}}_{2} ,{\text{b}}_{1} + {\text{b}}_{2} ,{\text{c}}_{1} + {\text{c}}_{2} )$$

4 $${\tilde{\text{e}}}_{1} - {\tilde{\text{e}}}_{2} = ({\text{a}}_{1} - {\text{a}}_{2} ,{\text{b}}_{1} - {\text{b}}_{2} ,{\text{c}}_{1} - {\text{c}}_{2} )$$

5 $${\tilde{\text{e}}}_{1} \otimes {\tilde{\text{e}}}_{2} = ({\text{a}}_{1} \times {\text{a}}_{2} ,{\text{b}}_{1} \times {\text{b}}_{2} ,{\text{c}}_{1} \times {\text{c}}_{2} )$$

6 $$\frac{{{\tilde{\text{e}}}_{1} }}{{{\tilde{\text{e}}}_{2} }} = \left( {\frac{{a_{1} }}{{c_{2} }},\frac{{b_{1} }}{{b_{2} }},\frac{{c_{1} }}{{a_{2} }}} \right)$$

7 $${\tilde{\text{e}}}_{1} \otimes {\text{k}} = ({\text{a}}_{1} \times {\text{k}},{\text{b}}_{1} \times {\text{k}},{\text{c}}_{1} \times {\text{k}})$$

### Fuzzy Delphi

Dalkey of RAND Corporation first founded the Delphi method. This method has been usually used in different studies, such as Azadeh et al. (2009), Rezgui et al. (2013), Mousavi et al. (2012). However, the traditional Delphi method cannot converge the vagueness and ambiguity information. Thus, Ishikawa et al. (1993) proposed FDM, and it was combined by the traditional Delphi technique and fuzzy set theory. Hsu et al. (2010) indicated that applying the FDM to group decision can solve the fuzziness of common understanding of decision-makers’ opinions. Concerning the selection of fuzzy membership functions, many studies were generally based on triangular fuzzy number, trapezoidal fuzzy number and Gaussian fuzzy number. This paper applies the triangular membership functions and the fuzzy theory to solve the group decision problems. This study uses FDM for the screening of alternate factors of the first stage. The fuzziness of common understanding of decision-makers could be solved by using the fuzzy theory, and evaluated on a more flexible scale. Moreover, the effectiveness and quality of questionnaires could be improved. Thus, more objective evaluation factors could be examined through the statistical results.

The FDM steps are as follows:

#### **Step 1:**

 Collection of opinions of decision group: Find the evaluation score of each criterion and alternative significances given by each decision-maker by using linguistic variables in questionnaires.

#### **Step 2:**

 Aggregation of group decisions matrices. The Fuzzy pairwise comparisons can be combined by using the following equation (Chang et al. 2009; Buyukozkan and Feyzıoglu 2004):

8 $$a_{ij} = \hbox{min} (a_{ijy} ),\quad b_{ij} = \left( {\prod\limits_{k = 1}^{k} {b_{ijy} } } \right)^{1/y} ,\quad c_{ij} = \hbox{max} (c_{ijy} )$$

where (a_ijy_, b_ijy_, c_ijy_) is the fuzzy evaluation of sample members ${\text{y}}\,\left( {{\text{y}} = 1,2, \ldots ,{\text{y}}} \right).$

### Fuzzy AHP

The AHP method developed by Saaty(1980) is widely used for tackling multi-criteria decision problems in real situations. However, in practice, crisp data are often inadequate to model many situations since human judgments are vague. To overcome classical AHP shortcomings, Van Laarhoven and Pedrycz (1983) proposed fuzzy AHP, which is the combination of AHP and fuzzy theory. Fuzzy AHP makes it possible to use linguistic ratings in the calculations of criteria weights by giving them a certain range. It is observed that decision-makers are more positive to give interval judgments than fixed-value judgments. Balli and Korukoğlu (2009) recognize that fuzziness in AHP contributes by being able to represent vague and ambiguity information.

There are many procedures for calculating the weights in fuzzy AHP technique proposed in the literature. Brief information about many of these procedures and a concise comparison can be found in Bozbura et al. (2007). In this study, the extent method introduced by Chang (1992) for handling fuzzy AHP, with the use of TFNs is used to calculate the fuzzy weights for the selected criteria (Chang 1992, 1996). The outlines of the Chang’s extent analysis method on fuzzy AHP have been explained in the following steps (Boutkhoum et al. 2015):

#### **Step 1:**

Fuzzy synthetic extent calculation

Let ${\text{X}} = \{ {\text{x}}_{1} ,{\text{x}}_{2} , \ldots ,{\text{x}}_{\text{n}} \}$ be an object set, and ${\text{G}} = \{ {\text{g}}_{1} ,{\text{g}}_{2} , \ldots ,{\text{g}}_{\text{m}} \}$ be a goal set. Using Chang’s extent analysis approach (Chang 1992, 1996), each object is taken on the extent analysis, for each goal, *g*_*i*_, is performed, respectively. Therefore, *m* extent analysis values for each object can be obtained, and are denoted as: ${\text{M}}_{\text{gi}}^{1} ,{\text{M}}_{\text{gi}}^{2} , \ldots ,{\text{M}}_{\text{gi}}^{\text{m}} ,\,{\text{i}} = 1,2,3, \ldots ,{\text{n}}$ where all the ${\text{M}}_{\text{gi}}^{\text{m}} ({\text{j}} = 1,2, \ldots ,{\text{m}})$ are TFNs.

With respect to the *i*th object, the value of fuzzy synthetic extent is defined as:

9 $${\text{S}}_{\text{i}} = \sum\limits_{{{\text{j}} = 1}}^{\text{m}} {{\text{M}}_{{{\text{g}}_{\text{i}} }}^{\text{j}} \otimes \left[ {\sum\limits_{{{\text{j}} = 1}}^{\text{n}} {\sum\limits_{{{\text{j}} = 1}}^{\text{m}} {{\text{M}}_{\text{gi}}^{\text{j}} } } } \right]^{ - 1} }$$

To obtain $\sum\nolimits_{{{\text{j}} = 1}}^{\text{m}} {M_{\text{gi}}^{\text{j}} }$ perform the fuzzy addition operation of m extent analysis values for a particular matrix such that

10 $$\sum\limits_{{{\text{j}} = 1}}^{\text{m}} {{\text{M}}_{\text{gi}}^{\text{j}} = \left( {\sum\limits_{{{\text{j}} = 1}}^{\text{m}} {{\text{a}}_{\text{j}} ,\sum\limits_{{{\text{j}} = 1}}^{\text{m}} {{\text{b}}{}_{\text{j}},\sum\limits_{{{\text{j}} = 1}}^{\text{m}} {{\text{c}}_{\text{j}} } } } } \right)}$$

And to obtain $\left[ {\sum\nolimits_{i = 1}^{n} {\sum\nolimits_{j = 1}^{m} {M_{{g_{i} }}^{j} } } } \right]{}^{ - 1}$ perform the fuzzy addition operation of ${\text{M}}_{\text{gi}}^{\text{m}} \,({\text{j}} = 1,2, \ldots ,{\text{m}})$ values such that

11 $$\sum\limits_{{{\text{i}} = 1}}^{\text{n}} {\sum\limits_{{{\text{j}} = 1}}^{\text{m}} {{\text{M}}_{\text{gi}}^{\text{j}} = \left( {\sum\limits_{{{\text{i}} = 1}}^{\text{n}} {{\text{a}}_{\text{i}} ,\sum\limits_{{{\text{i}} = 1}}^{\text{n}} {{\text{b}}_{\text{i}} ,\sum\limits_{{{\text{i}} = 1}}^{\text{n}} {{\text{c}}_{\text{i}} } } } } \right)} }$$

And then compute the inverse of the vector such that

12 $$\left[ {\sum\limits_{{{\text{i}} = 1}}^{\text{n}} {\sum\limits_{{{\text{j}} = 1}}^{\text{m}} {{\text{M}}_{\text{gi}}^{\text{j}} } } } \right]^{ - 1} = \left( {\frac{1}{{\sum\nolimits_{{{\text{i}} = 1}}^{\text{n}} {{\text{c}}_{\text{i}} } }},\frac{1}{{\sum\nolimits_{{{\text{i}} = 1}}^{\text{n}} {{\text{b}}_{\text{i}} } }},\frac{1}{{\sum\nolimits_{{{\text{i}} = 1}}^{\text{n}} {{\text{a}}_{\text{i}} } }}} \right)$$

#### **Step 2:**

 Comparison of fuzzy values

The degree of possibility of

M2 = (a_2_, b_2_, c_2_) ≥ M_1_ = (a_1_, b_1_, c_1_) is defined as:

13 $${\text{V}}\left( {{\text{M}}_{2} \ge {\text{M}}_{1} } \right) = \sup \left[ {\hbox{min} \left( {\upmu_{{{\text{M}}1}} ({\text{x}}),\upmu_{{{\text{M}}2}} ({\text{x}})} \right)} \right]$$

**Fig. 3:**
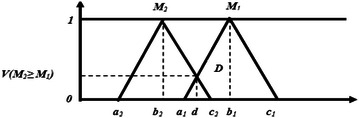
Intersection between M_1_ and M_2_

And can be equivalently expressed as follows:

14 $${\text{V}}({\text{M}}2 \ge {\text{M}}1) = {\text{hgt}}({\text{M}}1 \cap {\text{M}}2) \, = \left\{ {\begin{array}{*{20}l} {1,} \hfill &\quad {{\text{if}}\,b_{2} > b_{1} } \hfill \\ {0,} \hfill &\quad {{\text{if}}\,a_{1} \ge c_{2} } \hfill \\ {\frac{{a_{1} - c_{2} }}{{(b_{2} - c_{2} ) - (b_{1} - a_{1} )}},} \hfill &\quad {\text{Otherwise}} \hfill \\ \end{array} } \right.$$

where *d* is the ordinate of the highest intersection point *D* between ***μ***_***M1***_ and ***μ***_***M2***_ as shown in Fig. 3.

For the comparison of M_1_ and M_2_, we need both the values of V(M_1_ ≥ M_2_) and V(M_2_ ≥ M_1_).

#### **Step 3:**

 Priority weight calculation

The degree of possibility for a convex fuzzy number to be greater than *k* convex fuzzy numbers

${\text{M}}_{\text{i}} \,({\text{i}} = 1,2,3, \ldots ,{\text{k}})$ can be defined by:

15 $${\text{V}}\left( {{\text{M}} \ge {\text{M}}_{1} ,{\text{M}}_{2} , \ldots ,{\text{M}}_{\text{k}} } \right) = {\text{V}}\left[ {({\text{M}} \ge {\text{M}}_{1} )\,{\text{and}}\,({\text{M}} \ge {\text{M}}_{2} )\,{\text{and}} \ldots ({\text{M}} \ge {\text{M}}_{\text{k}} )} \right]$$

$${\text{V}}({\text{M}} \ge {\text{M}}_{1} ,{\text{M}}_{2} , \ldots ,{\text{M}}_{\text{k}} )$$ = min V (M ≥ M_i_), if

16 $${\text{m}}({\text{P}}_{\text{i}} ) = { \hbox{min} }\,{\text{V}}\left( {{\text{S}}_{\text{i}} \ge {\text{S}}_{\text{k}} } \right)\quad {\text{for}}\,{\text{k}} = 1,2, \ldots ,{\text{n;}}\quad {\text{k}} \ne {\text{i}}.$$

Then the weight vector is given by:

17 $${\text{W}}_{\text{p}} = \left( {{\text{m}}\left( {{\text{P}}_{1} } \right),{\text{m}}\left( {{\text{P}}_{2} } \right), \ldots ,{\text{m}}\left( {{\text{P}}_{\text{n}} } \right)} \right)^{\text{T}}$$

where P_i_$({\text{i}} = 1,2, \ldots ,{\text{n}})$ are n elements.

#### **Step 4:**

 Calculation of normalized weight vector

18 $${\text{W}} = \left( {{\text{W}}\left( {{\text{P}}_{1} } \right),{\text{W}}\left( {{\text{P}}_{2} } \right), \ldots ,{\text{W}}\left( {{\text{P}}_{\text{n}} } \right)} \right)^{\text{T}}$$

where *W* is a non-fuzzy number.

### Fuzzy TOPSIS

The TOPSIS technique is an MCDM method that was firstly proposed by Hwang and Yoon (1981), and Chen and Hwang (1992) later introduced a fuzzy TOPSIS. The basic concept of the fuzzy TOPSIS is to choose the alternative, which is as the shortest distance from the fuzzy positive ideal solution (FPIS) and longest distance from fuzzy negative ideal solution (FNIS). FPIS is a solution that maximizes the benefit criteria and minimizes cost criteria, whereas the FNIS maximizes the cost criteria and minimizes the benefit criteria (Wang and Elhag 2006). However, the fuzzy TOPSIS method is proposed where the weights of criteria and ratings of alternatives are evaluated by linguistic variables represented by fuzzy numbers (as shown in Table 2) and thereby allowing decision-makers to incorporate incomplete or vagueness information into the decision model (Önüt and Soner 2008; Kulak and Kahraman 2005; Korucu 2011).

**Table 2:** Linguistic variables for ratings

Linguistic scale	Triangular fuzzy number
Very bad (VB)	(0, 0, 1)
Bad (B)	(0, 1, 3)
Medium bad (MB)	(1, 3, 5)
Medium (M)	(3, 5, 7)
Medium good (MG)	(5, 7, 9)
Good (G)	(7, 9, 10)
Very good (VG)	(9, 10, 10)

In this paper, the overall priorities of the selected criteria are calculated using fuzzy AHP and the weights of criteria are used as input for ranking the alternatives by fuzzy TOPSIS. The general steps of fuzzy TOPSIS method can be described as follows (Chen 2000):

#### **Step 1:**

 Form a committee of decision-makers to rank the alternatives.

#### **Step 2:**

 Identify the evaluation criteria.

#### **Step 3:**

 Determine the appropriate linguistic variables for ranking alternatives with respect to selected criteria.

#### **Step 4:**

 Calculate the aggregated weight of alternatives with respect to each criterion (Kannan et al. 2014). If the fuzzy rating of all the decision-makers are described as TFNs.

Here, a = min_k_(a_k_), ${\text{b}} = \frac{1}{\text{k}}\sum\nolimits_{{{\text{k}} = 1}}^{\text{k}} {{\text{b}}_{\text{k}} }$, c = max_k_(c_k_). Then the aggregated fuzzy rating can be determined as ${\text{R}} = ({\text{a}},{\text{b}},{\text{c}}),\,{\text{k}} = 1,2,3, \ldots {\text{k}} .$

#### **Step 5:**

 Establish the fuzzy decision matrix as:

19 $${\tilde{\text{D}}} = \left[ {\begin{array}{*{20}c} {{\tilde{\text{x}}}_{11} } &\quad {\tilde{x}_{12} } &\quad \ldots &\quad {\tilde{x}_{{1{\text{n}}}} } \\ {{\tilde{\text{x}}}_{21} } &\quad {\tilde{x}_{22} } &\quad \ldots &\quad {\tilde{x}_{{2{\text{n}}}} } \\ \ldots &\quad \ldots &\quad \ldots &\quad \ldots \\ {{\tilde{\text{x}}}_{{{\text{m}}1}} } &\quad {\tilde{x}_{{{\text{m}}2}} } &\quad \ldots &\quad {\tilde{x}_{\text{mn}} } \\ \end{array} } \right]$$

Here, $${\tilde{\text{x}}}_{\text{ij}} = ({\text{a}}_{\text{ij}} ,{\text{b}}_{\text{ij}} ,{\text{c}}_{\text{ij}} )\;{\text{and}}\;{\tilde{\text{w}}}_{\text{j}} = ({\tilde{\text{w}}}_{{{\text{j}}{\mathbf{1}}}} ,{\tilde{\text{w}}}_{{{\text{j}}{\mathbf{2}}}} ,{\tilde{\text{w}}}_{{{\text{j}}{\mathbf{3}}}} );\;{\text{i}} = 1,2, \ldots ,{\text{m}},\;{\text{j}} = 1,2, \ldots ,{\text{n}}$$

Can be approximated by positive TFNs

#### **Step 6:**

 Normalized fuzzy decision matrix is indicated with *Ř* and expressed as in Eq. (19) (Chen 2000)

20 $${\tilde{\text{R}}} = \left[ {{\text{r}}_{\text{ij}} } \right]_{{{\text{m}} \times {\text{n}}}} \quad {\text{i}} = 1,2,3, \ldots ,{\text{m}};\quad {\text{j}} = 1,2,3, \ldots ,{\text{n}}$$

$${\text{where}}\,{\tilde{\text{r}}}_{\text{ij}} = \left( {\frac{{{\text{a}}_{{{\text{ij}}^{*} }} }}{{{\text{c}}_{\text{j}} }},\frac{{{\text{b}}_{{{\text{ij}}^{*} }} }}{{{\text{c}}_{\text{j}} }},\frac{{{\text{c}}_{\text{ij}} }}{{{\text{c}}_{\text{j}} }}} \right)\,{\text{and}}\;{\text{C}}_{\text{j}}^{ *} = \mathop {\hbox{max} }\nolimits_{i} {\text{C}}_{\text{ij}} .$$

#### **Step 7:**

 Construct weighted normalized fuzzy decision matrix.

Considering the different priorities value of each criterion, the weighted normalized fuzzy decision matrix is constructed as:

21 $${\tilde{\text{V}}} = \left[ {{\tilde{\text{v}}}_{\text{ij}} } \right]_{{{\text{m}} \times {\text{n}}}} \quad {\text{i}} = 1,2, \ldots ,{\text{m}}\quad {\text{and}}\;{\text{k}} = 1,2, \ldots ,{\text{n}}$$

${\tilde{\text{v}}}_{\text{ij}} = {\tilde{\text{r}}}_{\text{ij}} {\text{W}},$ where *W* is the weighted vector of evaluating criteria.

#### **Step 8:**

 Calculate the FPIS (A*) and FNIS (A−) as (Chen 2000):

22 $${\text{FPIS}}\left( {{\text{A}}^{*} } \right) = \left( {{\tilde{\text{V}}}_{1}^{*} ,{\tilde{\text{V}}}_{2}^{*} ,{\tilde{\text{V}}}_{3}^{*} , \ldots ,{\tilde{\text{V}}}_{n}^{*} } \right)\quad {\text{and}}\quad {\text{FNIS}}\left( {{\text{A}}^{ - } } \right) = \left( {{\tilde{\text{V}}}_{1}^{ - } ,{\tilde{\text{V}}}_{2}^{ - } ,{\tilde{\text{V}}}_{3}^{ - } , \ldots ,{\tilde{\text{V}}}_{\text{n}}^{ - } } \right)$$

$${\text{where}}\;{\tilde{\text{V}}}_{\text{i}}^{*} = \mathop {\hbox{max} }\nolimits_{\text{i}} \{ {\text{v}}_{\text{ijk}} \} \;{\text{and}}\;{\tilde{\text{V}}}_{\text{i}}^{ - } = \mathop{\min}\nolimits_{\text{i}} \{ {\text{v}}_{\text{ijk}} \} ;\,{\text{i}} = 1,2, \ldots ,{\text{m}};\,{\text{j}} = 1,2,3, \ldots ,{\text{n}}.$$

#### **Step 9:**

 Determine the distance of each alternative from FPIS and FNIS as

23 $$\begin{aligned} & {\text{d}}_{\text{i}}^{*} = \sum\limits_{{{\text{j}} = 1}}^{\text{n}} {{\text{d}}_{\text{v}} \left( {{\tilde{\text{V}}}_{\text{ij}} \times {\text{V}}_{\text{j}}^{*} } \right);\quad {\text{i}} = 1,2, \ldots ,{\text{m}}\quad {\text{and}}} \\ & {\text{d}}_{\text{i}}^{ - } = \sum\limits_{{{\text{j}} = 1}}^{\text{n}} {{\text{d}}_{\text{v}} \left( {{\tilde{\text{V}}}_{\text{ij}} \times {\text{V}}_{\text{j}}^{ - } } \right);\quad {\text{i}} = 1,2, \ldots ,{\text{m}}} \\ \end{aligned}$$

where *dv* is the distance measurement between two fuzzy numbers.

#### **Step 10:**

 Determine the closeness coefficient (*CC*_*i*_) for each alternative. The closeness coefficient represents the distances to the FPIS and FNIS for ranking the alternatives in descending order, it is calculated as (Chen 2000).

24 $${\text{CC}}_{\text{i}} = \frac{{{\text{d}}_{\text{i}}^{ - } }}{{{\text{d}}_{\text{i}}^{ - } + {\text{d}}_{\text{i}}^{*} }},\quad {\text{i}} = 1,2,3, \ldots ,{\text{m}}.$$

## FMCGDM framework

In today’s environment, it is very important for decision-makers to have access to the decision support tools in order to produce fast and accurate decisions. Many academic and commercial tools are available for the conventional multi-criteria decision-making methods as opposed to FMCGDM. However, these are far away from adjustment of our objective in incorporating fuzzy concepts into the analysis and using a specific prioritization procedure. Furthermore, it is essential to propose and create an appropriate decision support tool, which will include the power of web-based systems, group concepts, fuzzy concepts and empirical data, and thus improve the effectiveness of the prioritizing and classifying processes. The framework is designed as a web-based system since it is planned to be accessible from anywhere (Cakir and Canbolat 2008; Boroushaki and Malczewski 2010; Ma et al. 2010).

**Fig. 4:**
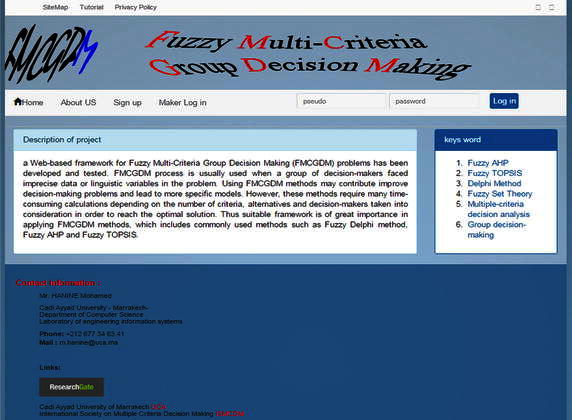
The home page of the FMCGDM framework

As indicated previously, application of FMCGDM methods may require important calculations. Furthermore, it is important for decision-makers to take into account different risk attitudes and scenarios. Also, an adequate framework is essential for appropriate and timely results. The developed web-based framework provides a tool for the necessary calculations and sensitivity analysis. It is developed by using PHP/Java Script Technology, MySql database, the Wamp web server and other open source software. Figure 4 shows the basic interface of the home page of this framework.

In this framework, it is supposed that users (administrators of projects) give inputs for the problem by filling different fields. For the application case, after registration, the administrator of the project should start with the description of the project, names of the criteria and sub-criteria, followed by the description of each alternative, and names and coordinates of each expert who participates in this evaluation process.

**Fig. 5:**
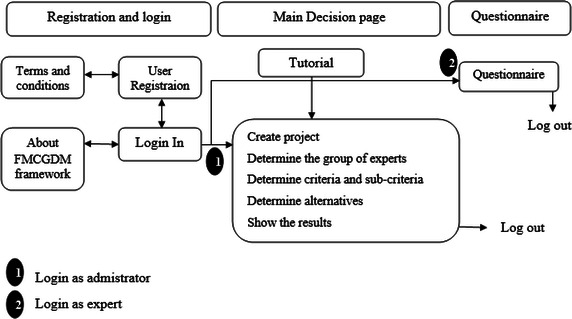
Sections and workflow of FMCGDM framework web site

Figure 5 indicates a basic flowchart for the workflow of FMCGDM framework, which is composed of three principal sections: (1) registration and log in, (2) the main decision page, and (3) the questionnaire. The registration and login section is composed of four windows: (1) log in as expert or as administrator, (2) user registration as administrator of a project, (3) terms and conditions, and (4) an “About FMCGDM framework” section. The users (administrators) accessing the Web site for the first time can register on the “Sign up” window after reading and agreeing to the terms and conditions. By completing the registration, users then are redirected to the Home page. Returning users can log into the system using the “Log In” Home page on which they are redirected to the “Main Decision page”. The “About FMCGDM framework” page introduces the design and development team.

**Fig. 6:**
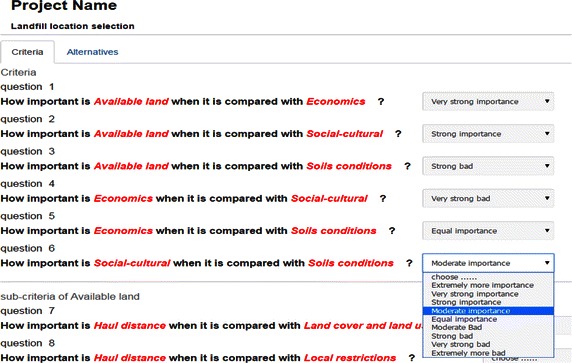
Example of questionnaire

Concerning the preferences of experts, users (as experts) are redirected to the questionnaire form page. The questionnaire facilitates the assessment of the different aspects of the project characteristics and provides data which can be used later for the usability assessment of the system. The collected data and information within the questionnaire form include many questions about comparing criteria, sub-criteria and other questions for comparing each alternative according to each criterion as presented in Fig. 6.

## Application of FMCGDM framework

FMCGDM framework is important through its usability and ability to form and perform analysis of alternatives and implement different methods on a single platform. It is an effective tool for multi-criteria analysis of a wide variety of scientific and practical problems.

This section presents an application of several tools, included in FMCGDM framework, for the analysis of the case study on landfill location selection in Casablanca. Casablanca covers a total area of 1156 km^2^ with a density of 3809 persons/km^2^. In this region, the increasing urbanization and the economic development lead to an increase in the quantity of generated solid waste. In fact, there are more than 500 active factories most of which are mostly related to energy, pharmaceuticals, foods, metal furniture, plastics and chemicals and there are relatively few phosphate derivatives, oil and aerospace factories.

The generation rate of solid waste of households amounts is approximately 3500 t/day, while the industrial solid waste represents more than 93,000 t/year, and the manufacturing of medical waste around 1030 t/year (Minenv 2013). However, because Casablanca is expected to grow, waste management strategy for the region should be re-evaluated. In the near future, there will be a need for a new landfill location to serve the region. Currently, four feasible locations (L1, L2, L3 and L4) are considered for landfilling. Moreover, six experts participate in this project for giving their opinions in the evaluation process. Based on the use of multi-criteria decision analysis for this problem, the evaluations of fourteen sub-criteria are further grouped into four main criteria as economy, available land, soils conditions and topography, and socio-cultural as presented in Table 3.

**Table 3:** The criteria and sub-criteria for selecting location of landfill municipal solid waste

Criteria	Sub-criteria
Economics criteria (C1)	Price of land (C11)
	Available transportation (C12)
	Effect on economic progress of surrounding region (C13)
	Infrastructure cost (C14)
	Land cover and land use (C21)
Available land (C2)	Haul distance (C22)
	Local restrictions (C23)
	Distance from rivers (C31)
Soils conditions and topography (C3)	Soil type (C32)
	Ground water quality (C33)
	Distance from wells (C34)
	Distance from residential areas(C41)
Social-cultural criteria (C4)	Distance from historical locations (C42)
	Wind direction (C43)

**Fig. 7:**
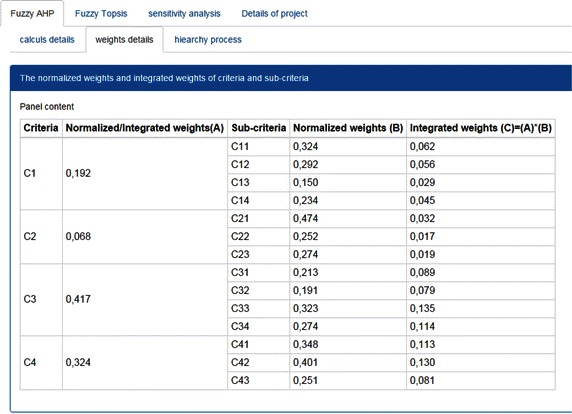
Results of the fuzzy AHP method

**Fig. 8:**
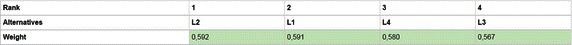
Results of the fuzzy TOPSIS method

After registration and filling the different fields as discussed above, each expert can Log into the framework to answer many questions about criteria, sub-criteria and alternatives as in Fig. 6. Initially the aggregation matrix of preferences of experts is performed and after, the weight coefficients of each criterion and sub-criterion are calculated after a set of comparison matrices by using the fuzzy AHP method (Fig. 7). The next step of the framework is dedicated to fuzzy TOPSIS to rank the alternatives. For this, the aggregation matrix of comparison alternatives according to each sub-criterion is carried out and after running of the process, global evaluations on alternatives are presented to the user, as shown in Fig. 8. The evaluation of all alternatives with the fuzzy TOPSIS analysis demonstrated that the ranking is determined as L2 > L1 > L4 > L3. The first alternative location (L2) is chosen as the best for landfill waste. In other words, the first alternative is closer to the FPIS and farther from the FNIS.

Additionally, the computation details are designed for demonstrating the different steps of computation for each method (fuzzy AHP and fuzzy TOPSIS) used in this framework. In FMCGDM framework, a performance table is carried out which encompasses not only the matrix of criteria against other criteria, but also against sub-criteria. Also, different steps of computing the weight coefficients of criteria and preferences alternatives are also presented.

Besides, tree structure is a tool incorporated in the framework for structuring the problem by developing a hierarchical set of criteria, sub-criteria and alternatives. The tree structure is also used for the representation of model data (criterion, sub-criteria scores for the determined alternatives, and weight coefficients).

**Fig. 9:**
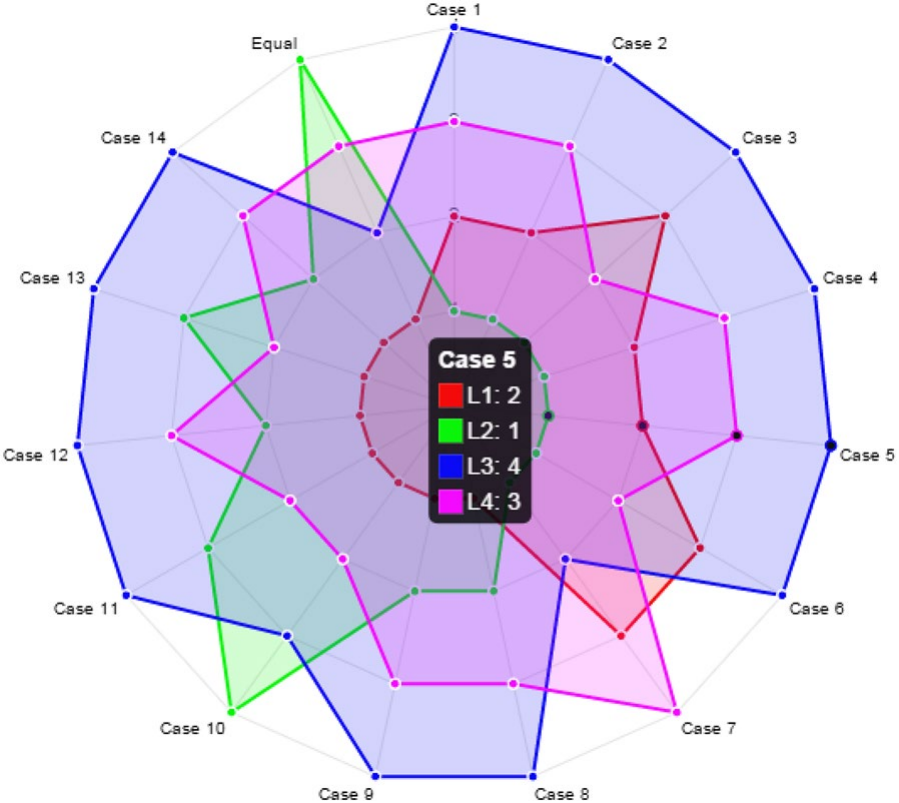
Graph of sensitivity analysis

The final step in the framework is to perform the sensitivity analysis to demonstrate the impact of the appreciation given by decision-makers on the selection of the best location for landfill solid waste. The sensitivity analysis in FMCGDM framework is performed by exchanging the results weights of criteria and sub-criteria. For analysis of multi-criteria problems, FMCGDM users can compare different alternatives according to the availability of corresponding data. Illustration of this step is presented in Fig. 9 with details of each case.

According to these results, it can be seen that the first case describes the original results of the present study. Also, out of fifteen cases, alternative location L3 has the lowest score in most cases. In the third case, the ranking of L1 is changing from 2 to 3. This also leads to change in ranking of L4 from 3 to 2. L2 and L1 are classified as first alternative locations in all cases considering the 4 criteria and 14 sub-criteria. Furthermore, the results of the sensitivity analysis indicate that the locations L2 and L1 are the most appropriate locations in all analyses for landfill of municipal solid waste. Also, this analysis makes the evaluation process easier for decision-makers.

## Conclusion and discussion

The purpose of this document is to describe the design of a new conceptual Web-based framework for FMCGDM. This framework is an application for analysis of multi-criteria problems. One of the fundamental differences of FMCGDM framework from other platforms/systems, illustrated in Table 1, is the inclusion of Delphi method a technique of groups of decision-making and fuzzy theory with popular multi-criteria decision making such as AHP and TOPSIS in a single framework. Therefore, FMCGDM framework allows uncertainty analysis via sensitivity analysis of the results to changing weight coefficients between sub-criteria.

The main reason behind the use of Delphi method for a group of decision-makers is that the method provides a mechanism for developing a constructive questionnaire, and transparent dialogue among decision-makers involved in the decision process rather than just supporting them in the identification of the optimal alternative. The FMCGDM framework is a web based system, which facilitates decision-making process for its users. For the implementation of this framework, we used PHP/Java Script Technology, MySql database, the Wamp web server and other open source software. We have illustrated the applicability of the framework through a case study of the landfill location selection. The proposed framework is flexible enough to fit other applications without changes and to incorporate different criteria in the evaluation process. The authors hope that this framework will enable practitioners/researchers to select, and evaluate many alternatives, using criteria that help them to make a better decision-support environment in their work systems. Additionally, the framework has been shown to be an effective platform for decision support when analyzing a wide range of multi-criteria problems.

The proposed prototype version has been demonstrated by a series of examples of multi-criteria decision-making problems in order to test the effectiveness of this framework. The survey is elaborated on the functions of this framework. Therefore, most members that have tested the framework are satisfied with functions of FMCGDM framework and encouraged for further work. For our forthcoming research, different techniques of MCDM, such as MACBETH, ELECTRE, VIKOR, TODIM, can be added to the FMCGDM framework and comparison of the results can be explored.
